# Effects of high-fat diet-induced obesity on circulating bone turnover markers in mice: a meta-analysis

**DOI:** 10.3389/fphys.2026.1875466

**Published:** 2026-09-11

**Authors:** Jie Ma, Binglin Chen, Bo Gao, Linyuan Zhang, Yuxiang Du

**Affiliations:** 1 Institute of Orthopedic Surgery, Xijing Hospital, Air force Medical University, Xi’an, Shaanxi, China; 2 The Second School of Clinical Medical College, Xuzhou Medical University, Xuzhou, Jiangsu, China; 3 School of Nursing, Air Force Medical University, Xi’an, Shanxi, China; 4 School of Exercise and Health, Shanghai University of Sport, Shanghai, China

**Keywords:** bone formation, bone resorption, meta-analysis, mouse model, obesity

## Abstract

**Objective:**

To investigate the level of bone turnover markers in the serum of mice induced into obesity by a high-fat diet, comparing them with non-obese counterparts.

**Data sources:**

PubMed, Cochrane Library, Embase, EBSCO, and Web of Science were searched from database inception to July 22, 2026.

**Methods:**

Two researchers screened literature based on inclusion and exclusion criteria, extracting valid data for quality evaluation. meta-analysis was conducted using Review Manager 5.4 software, focusing on primary outcomes related to blood biochemical markers associated with bone metabolism, such as TRAP, CTX-1, and OCN levels.

**Results:**

Seven studies were included in the systematic review, of which five reported sufficiently comparable data for inclusion in the meta-analysis. Five articles, encompassing data from 256 mice, met the inclusion criteria. Compared to the non-obese group, the serum of obese mice exhibited a significant increase in TRAP levels (MD=1.68 U/L, 95% CI=1.08-2.27, P<0.00001) as well as CTX-1 levels (MD=9.87 ng/mL, 95% CI=5.53-14.22, P<0.00001), with no statistically significant difference in OCN levels (SMD=0.26, 95% CI=-0.78-1.30, P=0.63).

**Conclusion:**

Current evidence from a limited number of animal studies suggests that high-fat diet-induced obesity may be associated with increased circulating levels of selected bone resorption markers, including TRAP and CTX-I, in mice. No statistically significant difference was observed for OCN. Given the small number of studies, methodological limitations, substantial heterogeneity in the OCN analysis, and the absence of quantitative structural skeletal outcomes, these findings should be considered preliminary.

## 1 Introduction

With the change in economic level and lifestyle, the worldwide prevalence of obesity has surged significantly, emerging as a pressing public health concern. Obesity is characterized by overt weight gain and the accumulation of a thick layer of body fat, primarily attributed to the excessive buildup of triglycerides. The World Health Organization (WHO) provides a standard for assessing obesity, categorizing Body Mass Index (BMI) into five grades, with a BMI exceeding 30 kg/m (Sabharwal and Root, 2012) deemed indicative of obesity (Sabharwal and Root, 2012; Caballero, 2019). Predictions suggest that by 2030, approximately 58% of the global adult population will be classified as overweight or obese (Vidal, 2002; Caballero, 2019).

Currently, there is a growing focus on the association between bone metabolism and metabolic diseases such as obesity, hyperlipidemia, and type 2 diabetes. Historically, scholars believed that obesity acted as a protective factor for bones. However, in recent years, an increasing number of studies have demonstrated that the relationship between obesity and bone health is complex (Głogowska-Szeląg et al., 2019).

The mechanical load on load-bearing bones such as the hip joint, femur, and lumbar spine is greater than that of non-load-bearing bones such as the upper limbs. Consequently, weight gain has a more pronounced impact on load-bearing bones, with obesity significantly increasing the bone mineral density (BMD) of the lumbar spine or total hip (Edelstein and Barrett-Connor, 1993; Rexhepi et al., 2015). We can further observe some interesting phenomena by researching animals. In a rat study, the BMD of the femur increased with a 20% weight gain but decreased when weight gain reached 34% or more (Li et al., 2017). Similarly, mice subjected to a weight gain of 31-34% experienced a decline in tibia BMD, whereas a weight gain of 17% did not yield such changes (Fujita et al., 2012). This indicates that the severity of obesity regulates the reduction of bone mass, but these changes require exceeding a certain threshold.

Bone turnover involves the continuous removal of old bone by osteoclast (bone resorption) and the ongoing production of new bone mineralized by osteoblast (bone formation). Detecting biochemical markers of bone metabolism helps in assessing bone turnover status. Despite numerous studies on the impact of a high-fat diet on bone metabolism globally, recent findings present conflicting conclusions. Therefore, this meta-analysis aimed to synthesize the available evidence on circulating bone turnover markers in mouse models of high-fat diet-induced obesity.

## 2 Materials and methods

This systematic review and meta-analysis adhered to PRISMA guidelines. The protocol for this study was registered on PROSPERO, and the registration number is CRD42022347986. The detailed record is accessible at https://www.crd.york.ac.uk/PROSPERO.

### 2.1 Search strategy

To identify all primary studies, we conducted searches across the following electronic databases: PubMed, EMBASE, Web of Science, Cochrane Library, and EBSCO. A uniform search strategy was devised employing the keywords “Obesity”, “Bone formation”, and “Bone resorption” across all databases. Detailed search strategies are outlined in the **Supplementary Table**. Additionally, we manually reviewed the reference lists included in the studies to identify other potentially relevant citations. No restrictions were imposed on language, publication date, or publication status.

### 2.2 Inclusion criteria for studies

#### 2.2.1 Types of studies

This analysis included controlled comparative animal studies on the effects of bone formation and bone resorption blood biochemical markers in a mouse model with high-fat diet-induced obesity.

#### 2.2.2 Types of animals

*In vivo* mice, irrespective of sex and age.

#### 2.2.3 Types of interventions

The intervention group was subjected to a high-fat diet without strict limitations on the composition, while the control group was exclusively fed a standard diet.

#### 2.2.4 Types of outcome measurements

The study focused on blood biochemical markers associated with bone metabolism, such as leptin, alkaline phosphatase (ALP), osteocalcin (OCN) and tartrate-resistant acid phosphatase (TRAP), osteoprotegerin (OPG), receptor activator of NF-κB ligand (RANKL), procollagen I C-Terminal propeptide (PICP), C-terminal telopeptide of type I collagen (CTX-1), N-terminal cross-linked peptide of type I collagen (NTX-1), N-terminal propeptide of type I collagen (PINP).

### 2.3 Study selection

Two researchers independently conducted a database search following the designated search strategies and imported all identified records into Endnote software. Evaluators included articles by removing duplications, screening titles and abstracts, and assessing full texts based on the inclusion criteria for studies. Any disagreements that arose during this process were resolved through discussion.

### 2.4 Data extraction

Following the full-text screening, two reviewers independently extracted data from each article and cross-verified the data of all included studies using a standardized data extraction table. The extracted information included general information such as the first author’s last name and year of publication, mouse characteristics (type, gender, and age), experimental model specifications, and details regarding high-fat intervention, including the high-fat component, number of interventions, and intervention time.

Furthermore, data on study findings and the risk of bias were also extracted. In cases where relevant data was not present in the article, we contacted the corresponding author to obtain the raw data. However, if there was no response, the Getdata software was used to retrieve the data shown in the chart. Any differences were resolved through discussion.

### 2.5 Assessment of risk of bias

The methodological quality of all included studies was assessed using the Systematic Review Center for Laboratory Animal Experimentation’s (SYRCLE) risk of bias tool for animal studies. This tool comprises 10 items reflecting six aspects of bias: (1) selection bias (sequence generation, baseline characteristics, and allocation concealment), (2) performance bias (random housing and blinding), (3) detection bias (random outcome assessment and blinding), (4) attrition bias (incomplete outcome data), (5) reporting bias (selective outcome reporting), and (6) other sources of bias. A response of “yes” indicates a low risk of bias, “no” indicates a high risk of bias, and “unclear” signifies insufficient details to measure the risk of bias.

### 2.6 Assessment of reporting bias

Publication bias was not formally assessed using funnel plots or statistical tests for funnel plot asymmetry because fewer than 10 studies contributed to each meta-analysis, resulting in insufficient statistical power and limited interpretability.

### 2.7 Statistical analysis

The meta-analysis was conducted using Review Manager software (RevMan 5.4; Cochrane Collaboration). Due to significant heterogeneity in the methods used for scoring across the included papers, random-effect models were employed for data synthesis. In instances where different tools measured the same results or different units of measurement were utilized in different studies, the standardized mean difference (SMD) was employed to combine continuous data during meta-analysis. Alternatively, the mean difference (MD) and a 95% confidence interval (CI) were used for analysis when uniform measurement tools were applied. Between-study heterogeneity was assessed using the Cochran’s Q-Test statistic (P < 0.1) and quantified with the I² statistic.

## 3 Results

### 3.1 Search results

**Figure 1:**
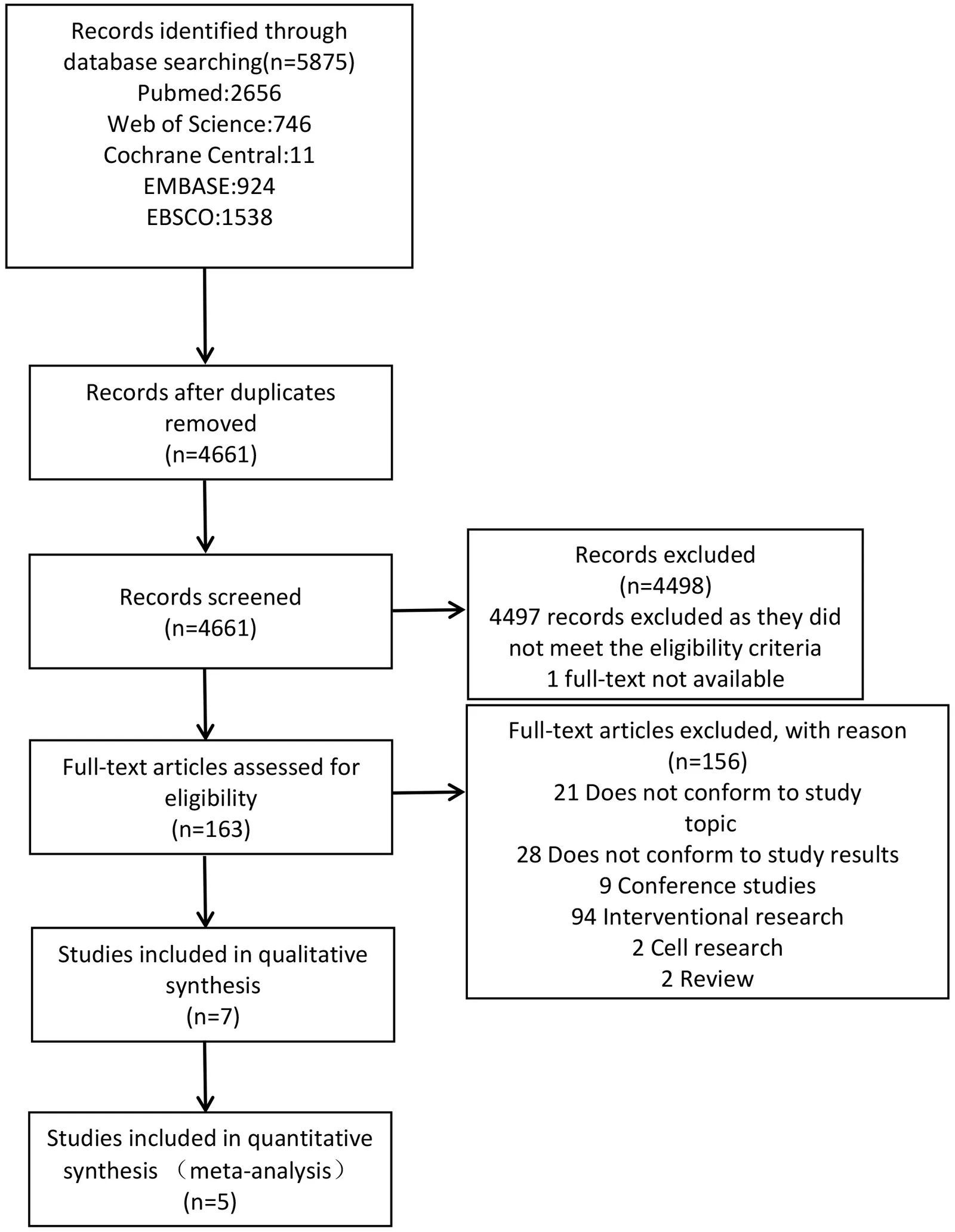
Flow chart of the study selection procedure.

A flow chart outlining the process of study identification and selection is depicted in **Figure 1**. Initially, we identified 5875 records through PubMed, EMBASE, Web of Science, Cochrane Library, and EBSCO. After eliminating duplicates, 4661 records remained. The researchers then preliminarily screened the records based on titles and abstracts, resulting in 163 records that underwent a comprehensive full-text review. During this review, 156 studies were excluded for various reasons, including being non-controlled studies, having outcomes outside the scope of our interest, providing only abstracts without full-text available, or lacking baseline information.

A total of seven studies met the eligibility criteria and were included in the systematic review. All seven studies were included in the descriptive synthesis and risk of bias assessment. Of these, five reported the prespecified outcome measures in a sufficiently comparable form and were included in the quantitative meta-analysis. Two studies were included in the narrative synthesis because they did not provide sufficient numerical data in a form suitable for effect-size calculation.

### 3.2 Study characteristics

**Table 1** summarizes the basic characteristics of the seven eligible studies, all of which were published between 2009 and 2022. The total sample size included 256 C57BL/6 mice, with 130 and 126 assigned to the high-fat diet and control groups, respectively. Four studies used only male mice (Cao et al., 2009; Cao et al., 2010; Xiao et al., 2010; Patsch et al., 2011), one study used only female mice (Lorincz et al., 2010), and two studies used both male and female mice (Gautam et al., 2014; Machnicki et al., 2022). Six studies specified the age at which the intervention commenced, ranging from 3 to 9 weeks, with a mean starting age of 5.5 weeks. All studies used a high-fat diet to induce obesity. The percentage of calories in the high-fat diet and control group varied, ranging from 21.2% to 60% and 4.3% to 10%, respectively. The number of mice per diet group exhibited variability, ranging from 6 to 57.

**Table 1:** Characteristics of included studies.

References	Species	Sex	Starting age (week)	Experimental model	% of kcal from fat in HFD groups, % (n)	% kcal from fat in CTR groups, % (n)	Duration of diet, weeks	Outcome
Machnicki et al., 2022	C57BL/6 mice	Male, Female	3	Diet	60%, n=57	10%, n=52	1,2,9 week	Leptin
Cao et al., 2010	C57BL/6 mice	Male	NR	Diet	45%, n=10	10%, n=11	14 week	TRAP, OCN, OPG, RANKL, Leptin
Xiao et al., 2010	C57BL/6 mice	Male	4	Diet	21.2%, n=8	4.8%, n=8	12 week	PICP, NTx
Lorincz et al., 2010	C57BL/6 mice	Female	9	Diet	39.5%, n=30	6%, n=30	10 week	TRAP, OCN
Cao et al., 2009	C57BL/6 mice	Male	6	Diet	45%, n=10	10%, n=11	14 week	TRAP
Patsch et al., 2011	C57BL/6 mice	Male	7	Diet	60%, n=12(sHF n=6; eHF n=6)	10%, n=6	LF: 24 week Shf: 21 week eHF:24 week	OCN, CTX-1, Leptin
Gautam et al., 2014	C57BL/6 mice	Male, Female	4	Diet	60%, F=8, M=8	4.3%, F=8, M=8	10 week	OCN, CTX-1

NR, No Reported; CTR, control diet; HFD, high-fat diet; sHF, short term high fat chow; eHF, extended high fat Chow.

In the six studies (Cao et al., 2009; Cao et al., 2010; Lorincz et al., 2010; Xiao et al., 2010; Patsch et al., 2011; Gautam et al., 2014) the intervention period lasted 10 weeks or longer. One study (Machnicki et al., 2022) featured three intervention periods of 1 week, 2 weeks and 9 weeks. The results from the included studies predominantly focused on blood biochemical indicators in mice. Leptin was assessed in three studies (Cao et al., 2010; Patsch et al., 2011; Machnicki et al., 2022), TRAP in three studies (Cao et al., 2009; Cao et al., 2010; Lorincz et al., 2010), OCN was in four studies (Cao et al., 2010; Lorincz et al., 2010; Patsch et al., 2011; Gautam et al., 2014), OPG in one study (Cao et al., 2009), RANKL was in one study (Cao et al., 2010), PICP in one study (Xiao et al., 2010), NTX in one study (Xiao et al., 2010), and CTX-1 in two studies (Patsch et al., 2011; Gautam et al., 2014).

### 3.3 Study quality assessment

The risk of bias judgments are summarized in **Table 2**. None of the included studies clearly described random sequence generation or allocation concealment; these domains were therefore judged as unclear risk of bias. Random housing was also insufficiently reported. Blinding of caregivers or investigators and blinding of outcome assessors were not adequately described and were judged as high or unclear risk according to the information reported in each study. Selective outcome reporting was generally judged as unclear because study protocols or prespecified analysis plans were unavailable. Overall, limited reporting of randomization, allocation concealment, blinding, and outcome handling reduced confidence in the internal validity of the included evidence.

**Table 2:** Risk of bias assessment.

SYRCLE’ s risk of bias tool for animal studies										
References	Sequence generation	Baseline characteristics	Allocation concealment	Random housing	Performance Blinding	Random outcome assessment	Blinding of outcome assessment	Incomplete outcome data	Selective outcome reporting	Other sources of bias
Machnicki et al., 2022	U	Y	U	U	N	U	N	N	U	Y
Cao et al., 2010	U	Y	U	U	N	U	N	N	U	Y
Xiao et al., 2010	U	Y	U	U	N	U	N	N	U	Y
Lorincz et al., 2010	U	Y	U	U	N	U	N	Y	U	Y
Cao et al., 2009)	U	Y	U	U	N	U	N	N	U	Y
Patsch et al., 2011	U	Y	U	U	N	U	N	Y	U	Y
Gautam et al., 2014	U	Y	U	U	N	U	N	Y	U	Y

Y, yes = low risk of bias; N, no = high risk of bias; U, unclear = unclear risk of bias.

#### 3.3.1 The effect of HFD on TRAP in serum

**Figure 2:**
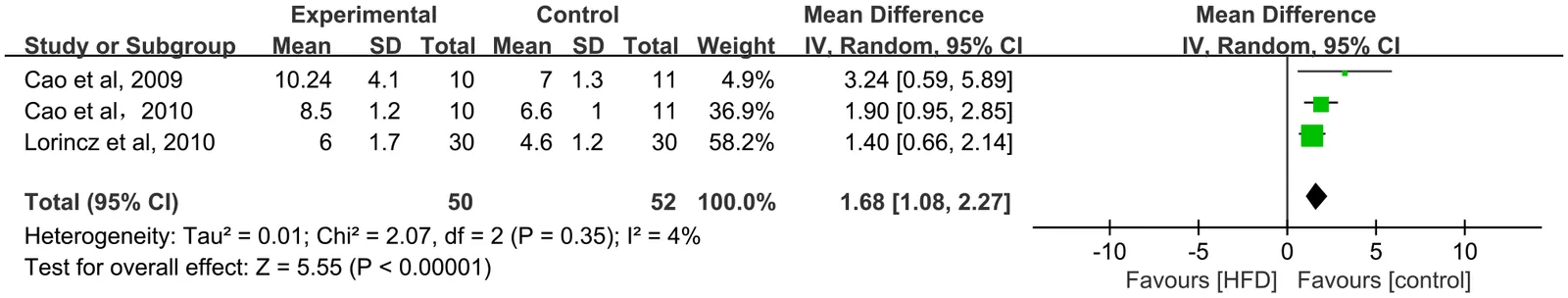
Forest plot of studies reporting data from TRAP.

Three articles (Cao et al., 2009; Cao et al., 2010; Lorincz et al., 2010) reported the effect of HFD on the level of TRAP. The study encompassed a total of 102 mice, with 50 mice in the HFD group and 52 in the control group. There was no significant heterogeneity among the studies (I (Sabharwal and Root, 2012) = 4, P=0.35). The pooled analysis showed significantly higher serum TRAP levels in the HFD group than in the control group (MD=1.68 U/L, 95% CI=1.08-2.27, P<0.00001). These results are shown in **Figure 2**.

#### 3.3.2 The effect of HFD on CTX-1 in serum

**Figure 3:**
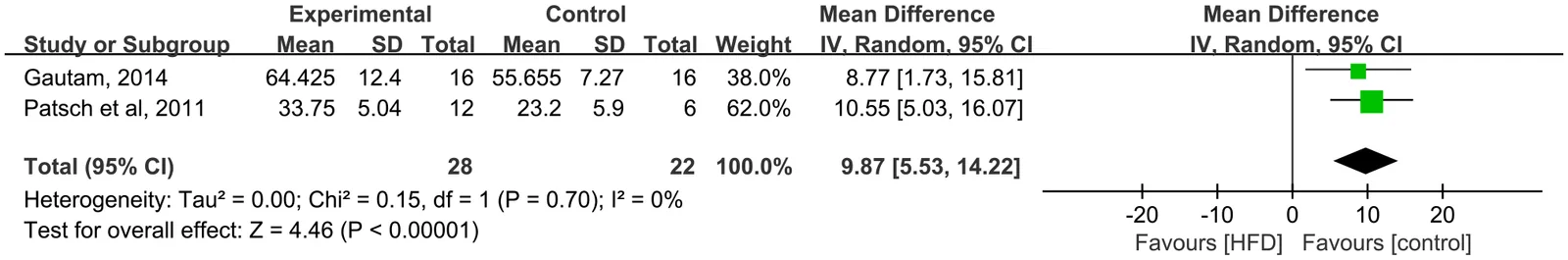
Forest plot of studies reporting data from CTX-1.

Two articles (Patsch et al., 2011; Gautam et al., 2014) studied the changes in serum bone biochemical index CTX-1. One study (Patsch et al., 2011) divided into two groups, one undergoing short-term high-fat intervention for 21 weeks and one undergoing long-term high-fat intervention for 24 weeks. The other study (Gautam et al., 2014) divided mice into two groups by sex: female and male. The total sample included 50 mice, with 28 in the high-fat group and 22 in the control group. No significant heterogeneity was observed among the studies (P=0.7, I^2^ = 0%). The pooled analysis showed significantly higher serum CTX-I levels in the HFD group than in the control group (MD=9.87 ng/mL, 95% CI=5.53-14.22, P<0.00001). These results are shown in **Figure 3**.

#### 3.3.3 The effect of HFD on OCN in serum

**Figure 4:**
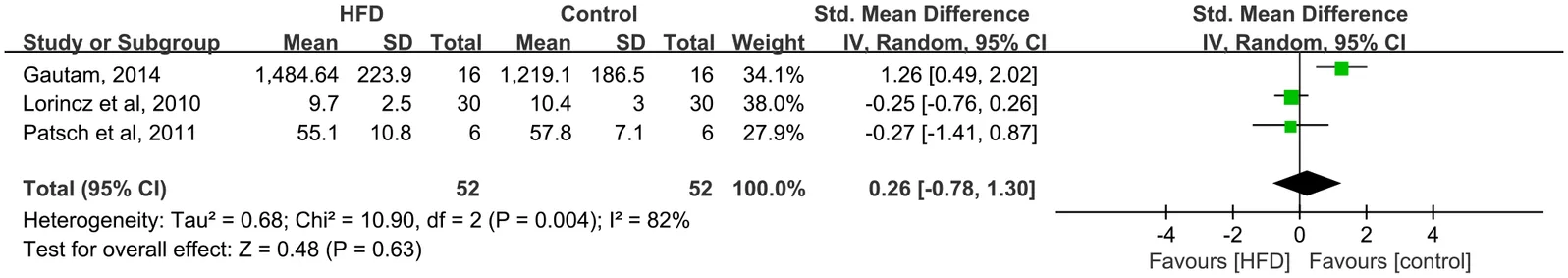
Forest plot of studies reporting data from OCN.

Three articles (Lorincz et al., 2010; Patsch et al., 2011; Gautam et al., 2014) studied the changes of bone biochemical index OCN in serum. one of which was further divided into two groups: female mice and male mice (Gautam et al., 2014). A total of 104 mice were included, including 52 mice in the high-fat group and 52 mice in the control group. There is significant heterogeneity among these studies (P= 0.004, I^2^ = 82%). The results of the standardized mean difference (SMD) indicated that No statistically significant between-group difference in OCN levels was observed. (SMD=0.26, 95% CI=-0.78-1.30, P=0.63). These results are shown in **Figure 4**.

## 4 Discussion

Seven studies were included in the systematic review. Five studies reported the prespecified outcome measures and were included in the meta-analysis, while two were synthesized narratively. The total sample consisted of 256 mice, comprising 130 obese mice induced by a high-fat diet and 126 normal control mice. The comprehensive analysis of all eight bone-related indexes (TRAP, CTX-1, OCN, Leptin, PICP, NTx, OPG, RANKL) revealed a significant increase in serum bone resorption markers in high-fat diet-induced obese mice. We utilized the Cochrane collaboration group recommendations to evaluate the risk of bias in each article. Notably, all articles demonstrated a high risk of other prejudices. Moreover, it is difficult to assess whether these articles accurately describe the intended outcome measures. In this meta-analysis, both the experimental and control groups were included in the study. Two studies (Xiao et al., 2010; Machnicki et al., 2022) were included in the narrative synthesis because they did not provide sufficient numerical data in a form suitable for effect-size calculation.

The C57BL/6 strain is commonly used in diet induced obesity studies because it is susceptible to metabolic alteration following high fat feeding (Kleinert et al., 2018). However, the severity of the obesity phenotype may depend on several experimental factors, including dietary fat content and intervention duration. Previous studies have shown that feeding mice with a 60% fat diet significantly increases the weight of obese mice, elevates serum triglyceride (TG), total cholesterol (TCH), and insulin (INS) levels, leading to a decline in glucose tolerance and insulin resistance (Al-Awar et al., 2016). Among the data we extracted, three articles (Patsch et al., 2011; Gautam et al., 2014; Machnicki et al., 2022) in the High-Fat Diet (HFD) group utilized a diet comprising 60% of kilocalories from fat, while two articles (Cao et al., 2009; Cao et al., 2010) employed a diet with 45% kilocalories from fat. The remaining two studies used diets with 21.2% and 39.5% fat, respectively (Lorincz et al., 2010; Xiao et al., 2010). Their control groups also employed significantly different fat contents. Of the control (CTR) groups, four utilized a diet with 10% kilocalories from fat (Cao et al., 2009; Cao et al., 2010; Patsch et al., 2011; Machnicki et al., 2022), and the other three used 4% to 6% (Lorincz et al., 2010; Xiao et al., 2010; Gautam et al., 2014). Such differences may have influenced the extent of weight gain and metabolic dysfunction and may consequently have affected bone turnover marker levels. Intervention duration also varied considerably across studies and may have further contributed to heterogeneity. Because only a small number of studies were available, these factors could not be evaluated reliably through subgroup analysis or meta-regression. Their effects on the pooled estimates therefore remain uncertain.

Bone metabolism, also known as bone remodeling or bone turnover, refers to the continuous renewal of bone mineral composition and bone matrix, including osteoclast-mediated bone resorption and osteoblast-mediated bone formation. Under normal circumstances, the amount of old bone absorbed by osteoclasts is consistent with the amount of new bone generated by osteoblasts. This equilibrium facilitates the continuous renewal of bone tissue, maintaining its mechanical integrity throughout life. This process plays an important role in maintaining the steady state of trace elements such as calcium and phosphorus in the body. However, under certain physiological or pathological conditions, the activities of osteoblasts and osteoclasts may be altered. If the two processes of bone metabolism become unbalanced, bone tissue undergoes corresponding changes, such as physiological growth and development, aging or pathological osteoporosis (OP), osteomalacia, bone metastasis, and so forth. At present, the widely used markers of bone formation include ALP, PINP, OCN, OPG, etc., and the markers of bone resorption include TRAP, CTX-1, NTX-1, hydroxyproline (HYP/HOP), and so forth (Chen et al., 2021).

### 4.1 Effect of HFD on serum bone formation markers

It is well-established that procollagen type I-derived PICP serves as an indicator of bone matrix formation and collagen type I synthesis. Consequently, the serum PICP level serves as a key indicator for assessing bone formation (Ying et al., 2023). Our results revealed only one retrieved article focusing on the expression of PICP in the serum of obese mice. Xiao et al. (2010) and other studies consistently demonstrated that the expression of PICP in the serum of obese mice induced by a high-fat diet was significantly lower than that in the control group. Osteoblasts promote the maturation of osteoclasts by releasing RANKL, which binds to RANK expressed by osteoclasts. At the same time, osteoblasts can inhibit the combination of RANK and RANKL by releasing osteoprotegerin (OPG), thereby achieving the opposite regulatory effect (Liu et al., 2021). When the RANKL/OPG ratio becomes imbalanced, it affects corresponding bone transformations, eventually leading to the corresponding bone system diseases. There is only one article addressing the expression of RANKL in the serum of obese mice. Cao et al. (2010) and other studies indicate that there is no significant difference in the expression of RANKL in the serum of obese mice when compared with the control group. Conversely, OPG exhibits the opposite pattern in one of the included articles, where its expression in the serum of obese mice is higher than that of the control group. Osteocalcin (OCN) is a non-collagen acidic glycoprotein synthesized and secreted by osteoblasts in bone. OCN is commonly used as a circulating marker of bone formation and is involved in bone matrix mineralization (Du et al., 2021). Accordingly, we quantitatively synthesized the available data on serum OCN levels. The pooled analysis showed no statistically significant differences in serum OCN levels between HFD group and controls. However, substantial heterogeneity was present in the OCN analysis (I2 = 82%).

Potential sources of heterogeneity included animal sex, developmental stage at the initiation of high-fat feeding, dietary fat content, and intervention duration. The included evidence comprised male-only, female-only, and mixed-sex studies, and one study reported combined data for male and female mice without providing sex-specific summary statistics. Because only three studies contributed to the OCN meta-analysis, subgroup analysis or meta-regression would have produced subgroups containing only one or two studies and were therefore not performed. In addition, these study-level characteristics varied concurrently, making it difficult to determine their independent contributions to heterogeneity.

Given the small number of studies, limited sample sizes, substantial heterogeneity, and methodological limitations, confidence in the pooled OCN estimate is limited. The finding should be considered preliminary and interpreted cautiously. Larger, rigorously designed animal studies using standardized dietary protocols, clearly defined animal characteristics, and consistent outcome-assessment methods are required to clarify the effect of high-fat diet-induced obesity on OCN and bone formation.

### 4.2 Effect of HFD on serum bone resorption markers

TRAP plays a crucial role in resisting tartaric acid inhibition, primarily originating from osteoclasts. TRAP, along with other isoenzymes, are involved in the degradation of solid calcium phosphate matrix within the bone matrix during bone resorption. As a specific marker enzyme for osteoclast differentiation and bone resorption function, TRAP serves as an indicator of osteoclast differentiation and maturity (Greenblatt et al., 2017). CTX-1 is the degradation product of type 1 collagen (COL-I), which can reflect the bone resorption. An increase in CTX-1 is indicative of enhanced osteoclast function (Chubb, 2012). In several of the included papers, it was found that the expression levels of TRAP and CTX-1 in the serum of obese mice were significantly increased following high-fat diet induction. The pooled analyses showed higher circulating TRAP and CTX-1 levels in HFD-induced obese mice. These findings may indicate increased bone resorption-related activity, but they do not establish structural bone loss or a generalized disturbance in bone remodeling. One included study also reported significantly higher serum NTx levels in obese mice than in controls. However, because this finding was derived from a single study, it should be interpreted cautiously and cannot be considered conclusive (Swarup et al., 2022). Leptin, initially identified in 1994, is a peptide hormone encoded by the ob/ob gene and is primarily synthesized and secreted by white adipocytes. As the earliest discovered adipocytokine, there exists a strong positive correlation between plasma leptin levels and body fat percentage (Obradovic et al., 2021). Currently, it is understood that the effect of leptin on bone metabolism is multifaceted, encompassing both peripheral and central functions. In the central pathway, leptin inhibits bone formation, promotes bone resorption, and consequently contributes to bone loss. In the peripheral pathway, leptin promotes bone formation and inhibits bone resorption, thus increasing bone mass (Wang et al., 2015). Among the articles we included, three specifically addressed the expression of leptin in the serum of obese mice (Cao et al., 2010; Patsch et al., 2011; Machnicki et al., 2022). Machnicki et al. (2022) and other revealed no significant difference in serum leptin expression levels between the two groups, while Patsch et al (Patsch et al., 2011) and Cao et al (Cao et al., 2010) showed that the expression of leptin in serum of obese mice was significantly higher than that of the control group. Because leptin was not quantitatively synthesized and the reported findings were inconsistent, no firm conclusion can be drawn regarding the association between circulating leptin and bone turnover in the included models. Moreover, circulating leptin levels do not directly reflect central leptin sensitivity or activation of downstream neural pathways. None of the included studies assessed hypothalamic leptin signaling, sympathetic nervous system activity, β2-adrenergic signaling, or osteoblast-specific responses. Therefore, the observed changes in bone turnover markers cannot be attributed to a central leptin-mediated mechanism based on the available evidence. Further studies combining circulating leptin measurements with direct evaluation of leptin signaling and structural skeletal outcomes are required.

Biochemical markers of bone metabolism are bone matrix components released into the bloodstream and urine through the intricate activities of osteoblasts and osteoclasts. The determination of these markers helps distinguish primary and secondary osteoporosis, categorize bone transformation types, predict bone loss rate, and assess fracture risk. However, it is important to acknowledge certain limitations, such as measurement instability and uncontrollable factors such as hormonal influences. To enhance clinical application, a collaborative approach with various imaging methods is recommended to ensure a more accurate diagnosis. In our meta-analysis, it was found that the expression level of bone formation marker PICP decreased in obese mice, while the expression level of OPG increased. However, there was no significant difference observed in the expression levels of RANKL and OCN. The quantitative synthesis showed higher TRAP and CTX-I levels in HFD-induced obese mice. Findings for leptin, NTx, PICP, OPG, and RANKL were derived from one or a small number of studies and should be considered descriptive.

## 5 Limitations

As far as we know, this marks the first systematic review and meta-analysis dedicated to the identification of biochemical markers associated with bone metabolism in the serum of a mice obesity model induced by a high-fat diet. A series of analytical techniques and quality evaluation methods were employed in the study to strictly evaluate the previous studies. The studies incorporated exhibited notable quality deficiencies, such as a small sample size, and lack crucial elements in research design and reporting, potentially compromising the authenticity and reliability of the results. In addition, significant heterogeneity was observed in the meta-analysis studies we included, and the source of this heterogeneity could not be further analyzed due to limitations of the included literature. The present analysis was limited to circulating bone turnover markers. None of the included studies provided extractable quantitative data on bone mineral density, micro-CT-derived trabecular or cortical parameters, or bone histomorphometry. Circulating biomarkers reflect aspects of bone formation and resorption but do not directly characterize bone mass, microarchitecture, tissue-level remodeling, or mechanical strength. Therefore, the present findings should not be interpreted as definitive evidence of structural skeletal deterioration. Future animal studies should integrate circulating biomarkers with micro-CT, bone mineral density, histomorphometric, and biomechanical assessments to provide a more comprehensive evaluation of the skeletal effects of high-fat diet-induced obesity. The internal validity of the evidence was also limited by methodological and reporting deficiencies in the included studies. None of the studies clearly described random sequence generation, allocation concealment, or blinded outcome assessment. These omissions increase uncertainty regarding selection and detection bias. Because the judgments were based on published reports, it remains possible that some procedures were performed but not reported. Nevertheless, insufficient reporting prevents a reliable evaluation of study rigor and reduces confidence in the pooled findings.

## 6 Conclusions

Current evidence from a limited number of animal studies suggests that high-fat diet-induced obesity may be associated with increased circulating levels of selected bone resorption markers, including TRAP and CTX-I, in mice. No statistically significant difference was observed in the bone formation marker OCN.

These findings provide preliminary evidence of altered bone turnover marker profiles, but they are insufficient to establish a generalized imbalance in bone remodeling, impaired osteoblast activity, increased osteoclast activity, or structural skeletal deterioration. Confidence in the pooled estimates is limited by the small number of studies, limited sample sizes, substantial heterogeneity in the OCN analysis, methodological limitations, and the absence of quantitative data on bone mineral density, microarchitecture, histomorphometry, and biomechanical properties. Further rigorously designed animal studies using standardized dietary protocols, clearly reported randomization and blinding procedures, and integrated biochemical, structural, histomorphometric, and biomechanical assessments are required to confirm these findings and clarify the skeletal effects of high-fat diet-induced obesity.
