# Supplementary material for: Effects of high-fat diet-induced obesity on circulating bone turnover markers in mice: a meta-analysis
Source: Front Physiol. 2026 Sep 11;17:1875466. doi: 10.3389/fphys.2026.1875466 (PMC13613331; doi:10.3389/fphys.2026.1875466)
Supplement: Supplementary file 1 [file SupplementaryFile1.docx]

Effects of high-fat diet-induced obesity on circulating bone turnover markers in mice: a meta-analysis

**Search strategies for all databases**

1. Search Strategy for Pubmed:

#1 "fatty"[Title/Abstract] OR "excess fat"[Title/Abstract] OR "body fat*"[Title/Abstract] OR "Obesity"[Title/Abstract] OR "Obesity"[MeSH Terms]

### #2 "Osteogenesis"[MeSH Terms] OR "osteogenesis*"[Title/Abstract] OR "Bone Formation"[Title/Abstract] OR "ossification*"[Title/Abstract] OR "bone metabolism"[Title/Abstract] OR "bone turnover"[Title/Abstract] OR "Alkaline Phosphatase"[MeSH Terms] OR "Alkaline Phosphatase"[Title/Abstract] OR "procollagen Type I N-terminal peptide"[Title/Abstract] OR "Osteocalcin"[MeSH Terms] OR "Osteocalcin"[Title/Abstract]

#3 "Bone Resorption"[MeSH Terms] OR "bone resorption*"[Title/Abstract] OR "osteoclastic*"[Title/Abstract] OR "Osteoclastogenesis"[Title/Abstract] OR "C-terminal telopeptides of type I collagen"[Title/Abstract] OR "Tartrate-Resistant Acid Phosphatase"[MeSH Terms] OR "Tartrate-Resistant Acid Phosphatase"[Title/Abstract] OR "deoxypyridinoline"[Title/Abstract]

#4 #2 OR #3

#5 #4 AND #1

#6 animals NOT humans

#7 #5 and #6

2. Search Strategy for web of science:

#1 TS=(fatty OR “excess fat” OR “body fat*” OR Obesity)

#2 TS=(Osteogenesis OR “Bone Formation” OR ossification* OR “bone metabolism” OR “bone turnover” OR “Alkaline Phosphatase” OR “procollagen Type I N-terminal peptide” OR Osteocalcin)

#3 TS=(“Bone Resorption” OR osteoclastic* OR Osteoclastogenesis OR “C-terminal telopeptides of type I collagen” OR “Tartrate-Resistant Acid Phosphatase” OR deoxypyridinoline)

#4 #2 OR #3

#5 #4 AND #1

#6 animals NOT humans

#7 #5 and #6

3. Search Strategy for Cochrane Library:

#1 MeSH descriptor: [Obesity] explode all trees OR ("fatty"):ti,ab,kw OR ("excess fat"):ti,ab,kw OR ("body fat*"):ti,ab,kw OR ("Obesity"):ti,ab,kw
#2 MeSH descriptor: [Osteogenesis] explode all trees OR MeSH descriptor: [Alkaline Phosphatase] explode all trees OR MeSH descriptor: [Osteocalcin] explode all trees OR (osteogenesis*):ti,ab,kw OR ("Bone Formation"):ti,ab,kw OR (ossification*):ti,ab,kw OR ("bone metabolism"):ti,ab,kw OR ("bone turnover"):ti,ab,kw OR ("Alkaline Phosphatase"):ti,ab,kw OR ("procollagen Type I N-terminal peptide"):ti,ab,kw OR (Osteocalcin):ti,ab,kw

#3 MeSH descriptor: [Bone Resorption] explode all trees OR MeSH descriptor: [Tartrate-Resistant Acid Phosphatase] explode all trees OR ("bone resorption*"):ti,ab,kw OR (osteoclastic*):ti,ab,kw OR (Osteoclastogenesis):ti,ab,kw OR ("C-terminal telopeptides of type I collagen"):ti,ab,kw OR ("Tartrate-Resistant Acid Phosphatase"):ti,ab,kw OR (deoxypyridinoline):ti,ab,kw

#4 #2 OR #3

#5 #4 AND #1

#6 animals NOT humans

#7 #5 and #6

4. Search Strategy for EMBASE:

#1 'fatty':ab,ti OR 'excess fat':ab,ti OR 'body fat*':ab,ti OR 'obesity':ab,ti OR 'obesity'/exp

#2 'osteogenesis'/exp OR 'osteogenesis*':ab,ti OR 'bone formation':ab,ti OR 'ossification*':ab,ti OR 'bone metabolism':ab,ti OR 'bone turnover':ab,ti OR 'alkaline phosphatase':ab,ti OR 'alkaline phosphatase'/exp OR 'procollagen type i n-terminal peptide':ab,ti OR 'osteocalcin':ab,ti OR 'osteocalcin'/exp

#3 'bone resorption'/exp OR 'bone resorption*':ab,ti OR 'osteoclastic*':ab,ti OR 'osteoclastogenesis':ab,ti OR 'c-terminal telopeptides of type i collagen':ab,ti OR 'tartrate-resistant acid phosphatase':ab,ti OR 'tartrate-resistant acid phosphatase'/exp OR 'deoxypyridinoline'/exp

#4 #2 OR #3

#5 #4 AND #1

#6 animals NOT humans

#7 #5 and #6

5. Search Strategy for Ebsco:

#1 SU Obesity OR AB fatty OR TI fatty OR AB excess fat OR TI excess fat OR AB body fat* OR TI body fat* OR AB Obesity OR TI Obesity

#2 SU Alkaline Phosphatase OR AB Alkaline Phosphatase OR TI Alkaline Phosphatase OR AB procollagen Type I N-terminal peptide OR TI procollagen Type I N-terminal peptide OR SU Osteocalcin OR TI Osteocalcin OR AB Osteocalcin OR SU Osteogenesis OR AB osteogenesis* OR TI osteogenesis* OR AB Bone Formation OR TI Bone Formation OR AB ossification* OR TI ossification* OR AB bone metabolism OR TI bone metabolism OR AB bone turnover OR TI bone turnover

#3 SU Bone Resorption OR AB Bone Resorption OR TI Bone Resorption OR AB osteoclastic* OR TI osteoclastic* OR AB Osteoclastogenesis OR TI Osteoclastogenesis OR AB C-terminal telopeptides of type I collagen OR TI C-terminal telopeptides of type I collagen OR SU Tartrate-Resistant Acid Phosphatase OR AB Tartrate-Resistant Acid Phosphatase OR TI Tartrate-Resistant Acid Phosphatase OR AB deoxypyridinoline OR TI deoxypyridinoline

#4 #2 OR #3

#5 #4 AND #1

#6 animals NOT humans

#7 #5and #6
